# Changes in the Composition and Surface Properties of Torrefied Conifer Cones

**DOI:** 10.3390/ma13245660

**Published:** 2020-12-11

**Authors:** Monika Aniszewska, Arkadiusz Gendek, Štěpán Hýsek, Jan Malaťák, Jan Velebil, Barbora Tamelová

**Affiliations:** 1 Department of Biosystems Engineering, Institute of Mechanical Engineering, Warsaw University of Life Sciences—SGGW, Nowoursynowska 164, 02-787 Warsaw, Poland; monika_aniszewska@sggw.edu.pl (M.A.); arkadiusz_gendek@sggw.edu.pl (A.G.); 2 Department of Wood Processing and Biomaterials, Faculty of Forestry and Wood Sciences, Czech University of Life Sciences Prague, Kamycka 129, Prague 6, 165 00 Prague, Czech Republic; hyseks@fld.czu.cz; 3 Department of Technological Equipments of Buildings, Faculty of Engineering, Czech University of Life Sciences Prague, Kamycka 129, Prague 6, 165 21 Prague, Czech Republic; malatak@tf.czu.cz (J.M.); tamelova@tf.czu.cz (B.T.); 4 Research Institute of Agricultural Engineering, Drnovska 507, Prague 6, 161 01 Prague, Czech Republic

**Keywords:** torrefaction, weight loss, lower heating value, elemental composition, SEM

## Abstract

The paper investigated the torrefaction of cones from three tree species: Scots pine (*Pinus sylvestris* L.), Norway spruce (*Picea abies* L.), and European larch (*Larix decidua* Mill.). The objective was to determine the effects of torrefaction temperature on the properties of cones with a view to their further use as a renewable energy source. Torrefaction was conducted at 200, 235, 275, and 320 °C for 60 min under an inert gas atmosphere. Elemental composition, ash content, and lower heating value (LHV) were measured for the original and torrefied samples. Torrefaction performance was evaluated using formulas for solid yield, higher heating value (HHV), HHV enhancement factor, as well as energy yield. Scanning electron microscopy (SEM) was used to assess elemental composition and structural changes at the surface of the torrefied material. For all the studied conifer species, the higher the torrefaction temperature, the greater the carbon and ash content and the higher the LHV (a maximum of 27.6 MJ·kg^−1^ was recorded for spruce and larch cones torrefied at 320 °C). SEM images showed that an increase in process temperature from 200 to 320 °C led to partial decomposition of the scale surface as a result of lignin degradation. Cone scales from all tree species revealed C, O, N, Mg, K, and Si at the surface (except for pine scales, which did not contain Si). Furthermore, the higher the temperature, the higher the enhancement factor and the lower the energy yield of the torrefied biomass. Under the experimental conditions, spruce cones were characterized by the lowest weight loss, the highest HHV, and the highest energy yield, and so they are deemed the best raw material for torrefaction among the studied species.

## 1. Introduction

Biomass is unique among renewable energy sources (RES) in that it can be applied both in the production of electrical energy and in the manufacture of chemical products [1,2]. Due to its widespread and decentralized availability (as compared to solar and wind energy), biomass is also the most popular type of RES [3]. On the other hand, its disadvantages include variable and inhomogeneous composition, high moisture content conducive to fungal proliferation, low bulk volume, and low energy density per unit of volume [2,3,4,5,6]. This entails energy expenditure on comminution, as well as transport and storage costs [7].

Torrefaction is conducted for a variety of plant materials, usually at 200–300 °C under an inert atmosphere [8]. In the available literature, this process has been described as an effective method of improving the quality of biomass and facilitating its applications in pyrolysis, such as destructive distillation, gasification, carbonization, and combustion [9]. The Web of Science database returns more than 2000 results for the query “torrefaction”, including more than 1000 papers concerning biomass torrefaction published over the past five years. Those studies mostly focus on the physicochemical properties of products and fuels [10,11,12,13], torrefaction modeling and kinetics [14,15], torrefaction use in conjunction with other processing methods [16,17,18], as well as technological and economic analyses [19,20]. Many authors have also reported on the benefits of biomass torrefaction, such as increased lower heating value (LHV), hydrophobicity, and improved comminution parameters [21,22,23,24,25].

The main objective of the torrefaction process is to obtain a higher energy density of biomass by its increasing carbon content while lowering the concentration of oxygen and hydrogen per unit of volume [9]. This goal is similar to charcoal production by carbonization, with the important difference being that the latter does not retain maximum energy in the product, and so leads to low energy yield.

Torrefaction changes the physical properties of material by compromising fiber strength, which may facilitate comminution and, thus, improve and accelerate biomass co-firing in coal-fired power plants. Thus, torrefaction may enable lower coal consumption and greater use of sustainable fuels without the need for additional installations [4].

Due to the complex structure of wood, which consists of cellulose, hemicellulose, and lignin components, its thermal degradation presents a complicated field of study. Three segments of weight loss curves may be distinguished depending on process temperature, with hemicellulose decomposing at 225–325 °C, cellulose at 305–375 °C, and lignin at 250–500 °C [26]. Torrefaction is conducted at 225–300 °C, which causes hemicellulose degradation [26,27,28], yielding an attractive product with increased energy density (as compared to raw material) that can be applied in gasification and co-firing.

Scots pine, Norway spruce, and European larch are the most widespread conifers in Europe used for seed harvesting. Aniszewska et al. [29] have reported that spent conifer cones (after seed extraction) have a high energy potential and that as much as 0.6–1.2 million tons of cones could be obtained annually in Poland. Harvesting such an amount might require high costs; however, tree cones as a by-product are also generated in seed extraction facilities, which in Poland, produce on average several tens of tons of dry processed cones annually. Thus, cones constitute a valuable RES that can be utilized either raw or after processing into improved fuels [30].

A review of the available literature revealed that the torrefaction of cones has not been investigated to date. The present study examined the effects of torrefaction temperature on the physical properties of cones to be used for energy purposes.

## 2. Materials and Methods

### 2.1. Materials

The study material consisted of Scots pine (*Pinus sylvestris* L.), Norway spruce (*Picea abies* L.), and European larch cones (*Larix decidua* Mill.) following seed extraction at the Czarna Białostocka facility (GPS: 53°18′21.7″ N 23°16′24.8″ E, in Białystok County, Podlaskie Voivodeship, Poland). The cones were collected from the Rytel (E 17°26′, N 53°48′, in Chojnice County, Pomeranian Voivodeship, Poland), Żednia (E 23°38′, N 52°58′, in Białystok County, Podlaskie Voivodeship, Poland), and Bielsk (E 23°06′, N 52°41′, Bielsk Podlaski, Bielsk County, Podlaskie Voivodeship, Poland) Forest Districts. The moisture content of spent cones was approx. 8%.

### 2.2. Torrefaction

The cones were torrefied at 200, 235, 275, and 320 °C, which roughly corresponds to the temperature range of 200–300 °C [9], which has been described and recommended in the literature for different materials [23,26,31,32,33,34,35,36,37]. Torrefaction was conducted at atmospheric pressure in a LECO TGA701 muffle furnace (LECO Corporation, St. Joseph, MI, USA) enabling the use of analytical gases. Torrefaction was conducted under an inert atmosphere at a constant nitrogen flow rate of 8.5 L·min^−1^. Each sample was placed in an aluminum foil and inserted into a ceramic crucible. The samples were first dried at 105 °C for 3 h, and subsequently nitrogen was introduced and they were heated at a rate of 10 °C∙min^−1^ up to the set temperature (200, 235, 275, or 320 °C), which was maintained over 60 min within ±1 °C. After torrefaction, the furnace cooled down spontaneously in the same atmosphere. The process was computer controlled, with the data recorded to the hard drive. Measurements were done at least in quintuplicate.

### 2.3. Elemental Analysis, Higher and Lower Heating Values, and Ash Content

For all samples, proximate and elemental composition, as well as heating values were determined. Moisture and ash content were measured using a LECO TGA701 thermo-gravimetric analyzer (LECO Corporation, USA) at 105 °C and 550 °C, respectively. The methods used corresponded to ISO 18134-3:2015 [38] for moisture and ISO 18122:2015 [39] for ash content.

Carbon (*C*), hydrogen (*H*), nitrogen (*N*), and sulfur (*S*) contents were determined using a LECO CHN628 + S analyzer (LECO Corporation, USA) using the manufacturer’s analysis method for biomass by combustion at 950 °C. The LECO flour standards were used to calibrate the instrument. For all samples, sulfur content was below the calibration range, i.e., 0.02% wt. Oxygen (*O*) content was calculated as a difference. Analysis results were converted to dry state according to ISO 16993:2016 [40].

A LECO AC600 bomb calorimeter (LECO Corporation, USA) was used for the determination of higher heating values (HHV), while lower heating values (LHV) were calculated according to ISO 18125:2017 [41].

The measurement procedure is described in detail in Piętka et al. [42].

### 2.4. Torrefaction Performance and Severity

Torrefaction performance [43] was evaluated by means of: solid yield, HHV, HHV enhancement factor, and energy yield. Solid yield was used to evaluate the effects of torrefaction on sample weight loss, which corresponds to energy yield [44,45], while the enhancement factor reflected the power output and energy density of the obtained bio-coal [21]. These parameters were calculated using the following Equations [2,46]:

(1) $$S_{y}=\frac{W_{torr}}{W_{raw}}\cdot 100\;\left[\%\right],$$

(2) $$E_{f}=\frac{HHV_{torr}}{HHV_{raw}},$$

(3) $$E_{y}=S_{y}\cdot E_{f}\;\left[\%\right],$$

where *S_y_*—solid yield (%), *E_f_*—enhancement factor, *E_y_*—energy yield (%), *W*—weight, *HHV*—higher heating value (MJ·kg^−1^), the subscripts “*raw*” and “*torr*” represent raw and torrefied biomass, respectively.

### 2.5. Scanning Electron Microscopy and Elemental Analysis

Scales taken from both reference (RAW) and torrefied cones [47] were analyzed in terms of elemental composition of their surface. Specimens for scanning electron microscopy (SEM) were manually peeled from cones, placed on pin stubs, and then gold-coated using a Q150R ES (Quorum Technologies, Lewes, UK). The applied device was a MIRA 3 SEM (TescanOrsay Holding, Brno, Czech Republic) with a secondary electron detector operated at 10 kV acceleration voltage, working distance of 15 mm, and magnifications of ×50, ×500, ×1000, and ×2000. The elemental composition of specimen surface was examined by an energy-dispersive spectroscopy system: A Bruker XFlash X-ray detector (Bruker, Billerica, MA, USA) and data processing software ESPRIT 2 from the same company. Hydrogen was not detectable by the method used. The images exported from SEM and elemental analysis were not modified in any way.

The elemental composition of the surface of individual scales was analyzed by means of SEM images processed with ImageJ software (version 1.53, developed by Wayne Rasband, available from https://imagej.net), which determined the numbers of pixels corresponding to specific colors and divided them by the overall number of pixels. A special macro was written whereby filters were used to define colors corresponding to the analyzed elements with RGB threshold values (±5%).

### 2.6. Statistical Analysis

Statistica v.13 software (TIBCO Software Inc., Palo Alto, CA, USA) was used to calculate the means and standard deviations of the measured parameters and to perform analysis of variance (ANOVA) comparing test variants at a significance level of α = 0.05.

## 3. Results and Discussion

### 3.1. Elemental Analysis and Heating Values

The results obtained for elemental composition, higher and lower heating values, and elemental composition are given in Table 1. In turn, Table 2 presents sample weight loss upon torrefaction at different temperatures for the three studied cone species.

Due to the high content of organic substances in plant materials, they mostly consist of organic carbon, which accounts for as much as 45–55% of their weight, depending on plant part and species. In the case of all the studied cone species, it was found that the higher the torrefaction temperature, the higher the percentage share of carbon and the lower the shares of oxygen and hydrogen, which is consistent with a previous study by Basu [9]. In the case of hydrogen, its percentage share in the reference sample and in samples torrefied at 200–275 °C was between 5.1–5.7%, which corresponds to the 5–9% range cited in the literature [48,49,50]. Samples exposed to 320 °C exhibited lower hydrogen levels (4.1–4.4%).

The greatest changes in elemental composition were observed between samples torrefied at 275 °C and 320 °C. The increment in carbon content ranged from 9.6% for spruce to 16% for pine. In turn, the decrease in the percentage share of oxygen was from 15.6% for pine to 9.8% for spruce, and that of hydrogen ranged from 1.0% for pine and larch to 0.6% for spruce. The shares of ash and nitrogen increased with temperature. In the case of ash content, the increments were 0.9% for pine, 1.6% for spruce, and 3.1% for larch as compared to the reference samples. Similar changes, including a considerable increase in ash content, up to 4% at a torrefaction temperature of 300 °C, have been reported by Gucho et al. [51]. At 320 °C, the increments in the percentage share of nitrogen amounted to 0.2% for pine, 0.6% for spruce, and 0.4% for larch with respect to the reference samples. According to literature data [49,50,52], the range of nitrogen content in forest material is very wide, from 0.1% to 6.5%. ANOVA revealed that the observed effects of torrefaction temperature changes on the relative proportions of C, H, N, and O in samples were statistically significant.

ANOVA (*p* < 0.05) also confirmed a significant effect of torrefaction temperature on LHV. In this study, LHV for reference samples ranged from 19.9 MJ·kg^−1^ for spruce to 20.3 MJ·kg^−1^ for pine to 20.6 MJ·kg^−1^ for larch. Similar, albeit somewhat lower, results for pine cones have been reported by Haykiri-Acma [53] (18.6 MJ·kg^−1^) and Kistler et al. [54] (17.6 MJ·kg^−1^), with the LHV of pine wood being usually 19.2–21.2 MJ·kg^−1^ [55]. The corresponding LHV levels of biomass torrefied at 320 °C increased significantly to 27.6 MJ·kg^−1^ (HHV—28.6 MJ·kg^−1^) for spruce, 26.8 MJ·kg^−1^ (HHV—27.7 MJ·kg^−1^) for pine, and 27.6 MJ·kg^−1^ (HHV—28.5 MJ·kg^−1^) for larch, with the increment ranging from 6.5 to 7.7 MJ·kg^−1^ with respect to the reference samples. This is in line with the studies of Chen et al. [16], (HHV 26.1–27.4 MJ·kg^−1^) and Gucho et al. [51] (26.8 MJ·kg^−1^). Thus, the obtained material is comparable to low quality coal, such as lignite characterized by HHV of approx. 27 MJ·kg^−1^.

At 200, 235, and 275 °C, the greatest percentage weight loss (Table 2) was obtained for spruce cones, and the lowest for pine cones. At 320 °C, the reverse was true: the highest weight loss was found for pine cones, and the lowest for spruce cones. The increase in sample weight loss between 200 °C and 320 °C was 19-fold for pine cones, nearly 11-fold for spruce, and over 15-fold for larch.

### 3.2. Torrefaction-Induced Changes in the Structure of Cone Scales

**Figure 1:**
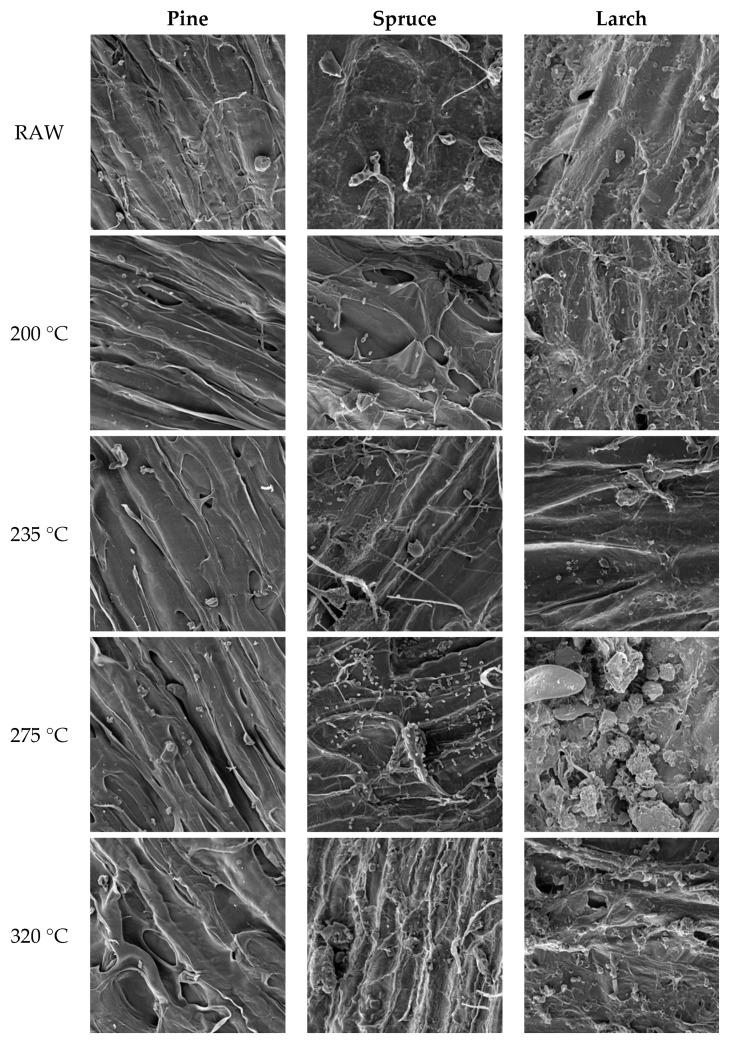
Example SEM images of the structure of pine scales, spruce scales, and larch scales for reference samples and for samples torrefied in the temperature range of 200–320 °C (magnification ×2000).

SEM images of cone scales of the three studied tree species taken from reference samples and from samples torrefied at the various temperatures, acquired at a magnification of ×2000, are presented in Figure 1.

SEM analysis revealed torrefaction-induced changes in scale structure, which became tubular. The number of openings in the scales increased with treatment temperature due to the volatile substances released during the process. This is consistent with the findings reported by Szwaja et al. [56] for Virginia mallow (*Sida hermaphrodita*).

The structures of pine, spruce, and larch scales taken from reference samples (Figure 1) differed markedly, with pine and larch scales exhibiting a fibrous appearance. Torrefaction at 200 °C altered those structures considerably, with the changes becoming even more pronounced with temperature increasing up to 320 °C. Processing in the temperature range of 200–320 °C led to the thermal decomposition of scale surface attributable to changes in hemicellulose content in the studied materials. According to Ibrahim et al. [3], in that temperature range sample weight loss is mostly caused by the release of water and gases by decomposing hemicellulose.

Cone scales torrefied at 200 °C (Figure 1) exhibited surface cracks and high porosity, with the structure observed at 235 °C being quite similar. Further major changes were observed upon torrefaction at 275 °C, especially for spruce and larch samples. As a result of hemicellulose and cellulose decomposition, the structure became tubular with spherical forms of various sizes. Finally, torrefaction at 320 °C led to partial degradation of scales, which is probably associated with the decomposition of lignin, which accounts for up to 60% of pine cone scales according to Aniszewska [57].

According to Ahmed et al. [47] and Wen et al. [58], hemicellulose decomposes at relatively low temperatures (below 200 °C), cellulose above 250 °C, and lignin above 300 °C (lignin degradation is slow and takes a long time) [59]. At temperatures above 250 °C, cell walls become increasingly thin as a result of gradual cellulose degradation. Examination of cone scales showed that above approx. 235 °C, the scale surface started to crack and the fibrous structure became compromised. Degradation changes in surface structure caused by a high degree torrefaction are very similar to degradation changes in the surface of lignocellulosic materials caused by enzymes [60].

After torrefaction, the cell wall structure of cone scales exhibited larger and damaged pores, which was associated with deeply collapsed cell walls. According to Chen et al. [61], this is attributable to the release of gases upon torrefaction. These changes may facilitate comminution as well as promote and accelerate reactions with other substances during gasification and combustion.

With increasing process temperature, the color of scales of the three cone species changed from brown to dark brown, and then to black. The temperature-induced color change was observed above 280 °C due to the exothermic nature of the reaction. When the process became exothermal, the produced heat increased the gas release rate. In turn, the higher amount of volatile substances facilitated the removal of oxygenated and hydrogenated compounds, leaving the solid torrefied product more concentrated in terms of fixed carbon [51]. Generally, high-carbon samples were darker, while after torrefaction at >300 °C, cone scales resembled charcoal; they also became less fibrous and more brittle, which may imply decreased energy consumption in the process of comminution.

### 3.3. Elemental Composition at the Surface of Cone Scales

Image analysis revealed the presence of six elements on the cone surface: carbon (C), oxygen (O), nitrogen (N), magnesium (Mg), potassium (K), and silicon (Si). Silicon was found in larch cones in the full temperature range and in spruce cones torrefied at 320 °C; it was absent from pine cones (Table 3).

**Figure 2:**
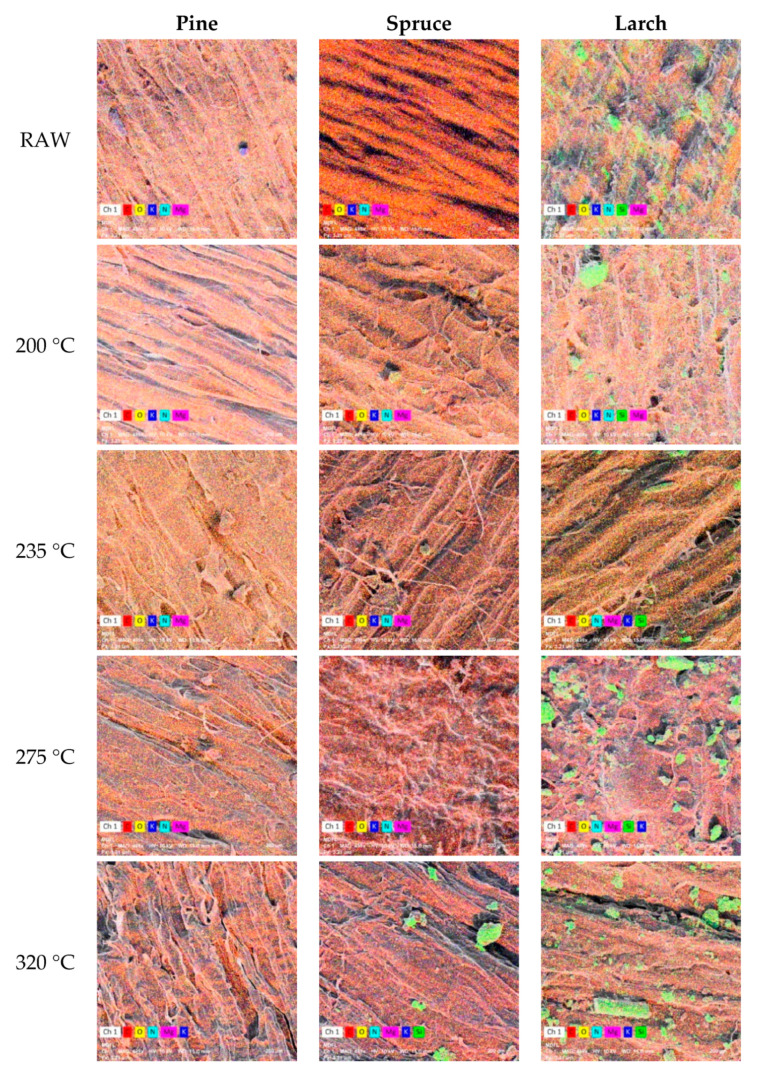
Examples of SEM images showing elemental composition of the surface of pine cone scales, spruce cone scales, and larch cone scales of reference and torrefied samples (magnification ×500). The color codes are as follows: *C*—red, *O*—yellow, *K*—dark blue, *N*—light blue, *Mg*—pink, *Si*—green.

Image analysis was used to evaluate the percentage composition of elements with the results given in Table 3. Sample images with elemental distribution at the surface of scales are presented in Figure 2. It was confirmed that carbon and oxygen have the highest percentage in lignocellulosic materials [62].

In the case of pine cone scales, the mean percentage share of carbon on the surface of reference samples was 60.2%. Torrefaction caused a decline in that element by approx. 11%, except for the samples exposed to 235 °C, where carbon content decreased to 45.1%. A similar situation was observed for magnesium and potassium, although their initial content in reference samples was much lower (26.9% Mg and 16.7% K, respectively). In the case of pine cone scales, the mean percentage share of carbon on the surface of reference samples was 60.2%. Torrefaction caused a decline in that element by approx. 11%, except for the samples exposed to 235 °C, where carbon content decreased to 45.1%. A similar situation was observed for magnesium and potassium, although their initial content in reference samples was much lower (26.9% Mg and 16.7% K, respectively).

After torrefaction at 235 °C, the percentage shares of Mg and K declined to 8.3 and 3.6%, to slightly increase after treatment at 320 °C (9.7 and 9.5%, respectively). Oxygen content at the surface of pine cone scales was 59.9% for the reference sample, and decreased to 26.6% at the highest process temperature. Finally, nitrogen content declined from an initial value of 7.1% to 1.8–1.6% following torrefaction at different temperatures (235, 275, and 320 °C).

The most abundant element at the surface of spruce cone scales was carbon, whose initial content of 71.9% decreased to 53.2% after torrefaction at 320 °C. At the same time, it should be noted that the percentage shares of carbon after treatments at 235 to 320 °C differed by only approx. 4%. The mean oxygen content for spruce scales amounted to 63.6%, which is approx. 3.7% more than for pine cone scales. Torrefaction at 275 °C decreased the percentage share of O to 19%. Nitrogen and magnesium content at the surface of spruce scales was on average 7.0 and 24.2%, similarly as in the case of pine scales; their percentage shares were halved upon torrefaction at 200 °C. The content of N reached a minimum of 1.2% at 275 °C, while the lowest content of Mg (5.4%) was observed at 235 °C. The mean percentage share of silicon at the surface of spruce scales torrefied at 320 °C was 5.9%.

As compared to the other two studied cone species, the surface of larch cone scales revealed the lowest levels of C, O, and N. The content of Mg was similar to that for spruce scales, while that of K was greater. The high percentage share of Si observed at the surface of reference larch samples (24.4%) declined to 5.0% at 235 °C and 1.2% at 320 °C.

No significant differences in carbon content were noted for reference larch and spruce samples torrefied at 200 °C and 235 °C (*p* = 0.431), in contrast to pine samples (*p* < 0.05). Significant differences were not found for material torrefied at 275 and 320 °C (*p* > 0.104).

Statistically significant differences (*p* < 0.001) in the content of K at the surface of scales were observed for reference samples torrefied at 200 and 320 °C. No significant differences were found for pine and larch cones treated at 235 °C (*p* = 0.567), and for spruce and pine cones exposed to 275 °C (*p* = 0.856).

The mean content of Mg at the surface of cone scales differed significantly for all samples torrefied at 200 and 320 °C (*p* < 0.05), but not for larch and spruce reference samples torrefied at 235 °C (*p* = 0.994 and *p* = 0.087) or for pine and spruce samples exposed to 275 °C (*p* = 0.472).

In the case of N, no significant differences in mean percentage shares were found for the three cone species torrefied at 235 and 320 °C. Significant differences in the content of this element were found at 200 °C (*p* < 0.002). In the case of samples treated at 275 °C (*p* = 0.135) and reference samples, it was found that the cone species did not affect the share of that element only for pine and spruce samples (*p* = 0.918).

The content of O on the surface of reference samples did not differ significantly between pine and spruce (*p* = 0.104). After torrefaction at 200 °C, significant differences were found only for larch and spruce samples (*p* = 0.022), while in the case of samples torrefied at 235 °C and 320 °C significant differences were shown for all three species (*p* < 0.001 and *p* < 0.025). At 275 °C no significant differences in O content on scale surface were identified for larch and pine (*p* = 0.238), while for the other samples significant differences did occur.

### 3.4. Torrefaction Performance Indicators

Table 4 presents results for solid yield, *S_y_* computed from Equation (1), enhancement factor, *E_f_* (Equation (2)), and energy yield, *E_y_* (Equation (3)).

The results show that *S_y_* decreased with increasing torrefaction temperature. The lowest percentage value of *S_y_* was found for pine cones (49.8%), and the highest for spruce cones (53.4%) torrefied at 320 °C, which means that approx. 47–50% wt. of the biomass is thermally degraded as compared to only approx. 2.5–4.3% wt. at 200 °C. Those losses may be correlated with ash content for the various species, with the lowest levels found for pine cones (implying greater organic content). As spruce and larch cones had slightly higher ash content, they exhibited a lower weight loss. These findings are consistent with the report of Zhang et al. [46].

In the temperature range 200–300 °C, the loss of sample weight was mostly attributable to the release of water and gases due to decomposing hemicellulose. According to Cai et al. [63], the release of volatile products during torrefaction degrades the smooth and homogeneous surface of the treated material.

The value of *E_f_* increased from 0.98 for the reference pine and spruce samples to 1.35 for spruce cones torrefied at 320 °C, which is consistent with the report of Zhang et al. [46]. 

**Figure 3:**
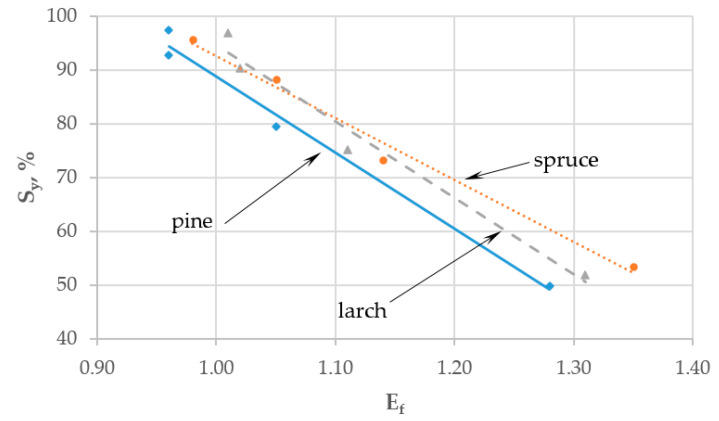
Linear relationships between solid yield (*S_y_*) and enhancement factor (*E_f_*) for torrefied cones from three tree species.

Linear relationships between *S_y_* and *E_f_* for the three torrefied cone species are given in Figure 3 and described with the following Equations:

(4) $$S_{yp}=-141.02\cdot E_{fp}+229.69\;r=0.988$$

(5) $$S_{yl}=-141.74\cdot E_{fl}+236.26\;r=0.973$$

(6) $$S_{ys}=-115.37\cdot E_{fs}+208.02\;r=0.987$$

with the subscript letters meaning: *p*—pine, *l*—larch, *s*—spruce.

A 0.1 increment in *E_f_* was found to decrease *S_y_* by 11.5–14% at high correlation coefficients (>0.986). The percentage value of *E_y_* declined from 98% for the reference sample of larch cones to 63.7% for torrefied pine cones (320 °C). Percentage differences in *E_y_* were approx. 30% for pine and spruce cones and 21.9% for spruce cones as compared to the other two species.

## 4. Conclusions

The main factor affecting torrefaction is process temperature, which is crucial in terms of obtaining the optimum amount of energy. The study shows that conifer cones from the studied three species of forest trees constitute a good renewable material for torrefaction and have substantial potential for use in energy conversion technologies.

Upon the torrefaction of samples under laboratory conditions, their lower heating value increased with process temperature, to reach 26.8 MJ·kg^−1^ for pine and 27.6 MJ·kg^−1^ for spruce and larch at 320 °C, which corresponds to LHV levels for lignite.

The higher the torrefaction temperature, the higher the general energy density and the lower the solid and energy yield. Under the experimental conditions, spruce cones exhibited the smallest weight loss, the highest HHV/LHV ratio, and the greatest energy yield, and so they can be deemed the best material for the production of torrefied biomass.

The observed torrefaction-induced changes to the scale structure at different temperatures mean that the cones of all the three studied species became more brittle, and so their comminution in subsequent processing stages would require less energy.

The presented findings considerably extended knowledge about the torrefaction of plant biomass. The quality of the obtained bio-coals was improved as compared to the raw material. To maximize torrefaction benefits while maintaining high energy yield, the whole process must be carefully optimized, which necessitates further study.

## Figures and Tables

**Table 1:** Elemental composition, ash content, higher heating value (HHV), and lower heating value (LHV) in dry state.

Temp.	C	H	N	S	O	Ash	HHV	LHV
°C			%				MJ·kg^−1^	
Pine								
RAW	54.9 ± 1.9	5.6 ± 0.3	0.1 ± 0.06	<0.02	38.6 ± 1.8	0.6 ± 0.1	21.6 ± 0.1 ^a^	20.3
200	53.9 ± 0.8	5.5 ± 0.1	0.1 ± 0.03	<0.02	39.7 ± 0.8	0.7 ± 0.2	20.8 ± 0.3 ^a^	19.6
235	55.6 ± 0.8	5.4 ± 0.1	0.1 ± 0.05	<0.02	38.0 ± 0.8	0.7 ± 0.2	20.9 ± 0.2 ^a^	19.7
275	60.4 ± 0.6	5.1 ± 0.1	0.2 ± 0.02	<0.02	33.4 ± 0.6	0.9 ± 0.2	22.8 ± 0.0 ^a^	21.7
320	76.4 ± 0.2	4.1 ± 0.0	0.3 ± 0.04	<0.02	17.8 ± 0.2	1.5 ± 0.1	27.7 ± 1.7 ^b^	26.8
Spruce								
RAW	54.0 ± 0.9	5.7 ± 0.1	0.8 ± 0.09	<0.02	37.6 ± 1.0	1.9 ± 0.1	21.1 ± 0.1 ^a^	19.9
200	54.9 ± 0.5	5.5 ± 0.1	0.9 ± 0.06	<0.02	36.4 ± 0.5	2.2 ± 0.4	20.8 ± 0.0 ^a^	19.6
235	58.7 ± 1.2	5.5 ± 0.1	1.0 ± 0.13	<0.02	32.6 ± 1.3	2.3 ± 0.2	22.1 ± 0.0 ^b^	20.9
275	63.7 ± 0.6	5.0 ± 0.1	1.1 ± 0.15	<0.02	27.4 ± 0.8	2.8 ± 0.1	24.1 ± 0.1 ^c^	23.0
320	73.3 ± 0.3	4.4 ± 0.0	1.4 ± 0.07	<0.02	17.6 ± 0.3	3.5 ± 0.3	28.6 ± 0.3 ^d^	27.6
Larch								
RAW	51.9 ± 0.3	5.7 ± 0.1	0.8 ± 0.20	<0.02	39.8 ± 0.1	1.7 ± 0.3	21.9 ± 0.0 ^a^	20.6
200	52.8 ± 0.3	5.7 ± 0.1	0.8 ± 0.31	<0.02	38.7 ± 0.3	2.0 ± 0.1	22.1 ± 0.2 ^a^	20.9
235	54.9 ± 0.4	5.6 ± 0.1	1.0 ± 0.38	<0.02	36.7 ± 0.1	1.9 ± 0.1	22.4 ± 0.0 ^a^	21.1
275	60.3 ± 0.7	5.1 ± 0.0	1.0 ± 0.26	<0.02	31.3 ± 0.8	2.4 ± 0.4	24.2 ± 0.0 ^b^	23.1
320	70.6 ± 1.5	4.1 ± 0.1	1.2 ± 0.08	<0.02	19.3 ± 1.5	4.8 ± 0.5	28.5 ± 0.1 ^c^	27.6

Note: Mean (±standard deviation); ^a,b,c,d^—the same letters indicate homogenous groups at *p* = 0.05.

**Table 2:** Sample weight loss (%) depending on torrefaction temperature.

Temp., °C	Pine	Spruce	Larch
200	2.6 ± 0.4	4.3 ± 0.1	3.1 ± 0.2
235	7.2 ± 0.4	11.8 ± 0.4	9.6 ± 0.4
275	20.1 ± 0.9	26.7 ± 0.7	24.9 ± 2.8
320	50.2 ± 0.3	46.6 ± 0.7	48.1 ± 0.6

**Table 3:** Mean percentage shares (%) of individual elements at the surface of cones torrefied at 200–320 °C (RAW—raw material).

Species	Temp.	C	O	N	Mg	K	Si
	°C	%					
Pine	RAW	60.2 ± 10.6	59.9 ± 6.3	7.1 ± 1.2	26.9 ± 2.3	16.7 ± 2.9	-
	200	58.8 ± 3.5	56.5 ± 3.5	5.0 ± 1.0	24.2 ± 2.1	18.8 ± 2.4	-
	235	45.1 ± 5.1	51.0 ± 2.9	1.8 ± 0.7	8.3 ± 1.9	3.6± 1.8	-
	275	53.3 ± 3.6	30.1 ± 2.0	1.7 ± 0.9	9.1 ± 2.1	7.0 ± 2.6	-
	320	49.2 ± 10.1	26.6 ± 4.3	1.6 ± 0.8	9.7 ± 2.1	9.5 ± 2.9	-
Spruce	RAW	71.9 ± 3.4	63.6 ± 3.3	7.0 ± 1.1	24.2 ± 2.0	22.0 ± 2.7	-
	200	69.8 ± 6.4	59.4 ± 4.3	3.3 ± 1.1	13.5 ± 2.8	9.3 ± 2.5	-
	235	55.3 ± 3.0	24.5 ± 2.4	1.3 ± 0.7	5.4 ± 1.7	5.8 ± 2.5	-
	275	57.0 ± 6.3	19.0 ± 2.7	1.2 ± 0.7	8.5 ± 3.3	6.8 ± 2.7	-
	320	53.2 ± 11.0	20.7 ± 1.9	1.4 ± 0.7	7.7 ± 2.0	19.9 ± 2.7	5.9 ± 1.4
Larch	RAW	48.2 ± 9.2	48.6 ± 7.7	6.0 ± 1.4	24.2 ± 3.4	29.7 ± 3.3	24.4 ± 3.4
	200	74.0 ± 7.0	54.8 ± 6.6	8.4 ± 2.2	27.9 ± 3.3	23.2 ± 2.8	10.2 ± 2.2
	235	53.8 ± 6.1	30.9 ± 3.7	1.4 ± 0.8	4.2 ± 1.7	4.0 ± 1.9	5.0 ± 0.7
	275	54.7 ± 9.0	29.1 ± 2.6	4.1 ± 1.3	18.0 ± 2.0	19.0 ± 2.5	10.8 ± 2.4
	320	54.9 ± 4.3	23.3 ± 2.5	1.4 ± 0.7	5.6 ± 1.8	6.1 ± 2.0	13.2 ± 2.4

**Table 4:** Torrefaction performance indicators.

Temp., °C	Pine	Spruce	Larch
***S_y_*, %**			
200	97.4 ± 0.4	95.7 ± 0.1	96.9 ± 0.2
235	92.8 ± 0.4	88.2± 0.4	90.4 ± 0.4
275	79.4 ± 0.9	73.3± 0.7	75.1 ± 2.8
320	49.8± 0.3	53.4 ± 0.7	51.9 ± 0.6
***E_f_*, -**			
200	0.98	0.98	1.01
235	0.96	1.05	1.02
275	1.05	1.14	1.11
320	1.28	1.35	1.31
***E_y_*, %**			
200	93.7	94.1	98.0
235	89.3	92.5	92.5
275	83.7	83.7	83.0
320	63.7	72.2	67.8
